# Institutional Appliances

**Published:** 1917-08-25

**Authors:** 


### Institutional Appliances.

### MODERN STEAM DISINFECTION.

We have received from the Grampian Engineering Company, Ltd., of 43 Aldwych, W.C., an instructive and well-illustrated pamphlet describing the "Velox" high-pressure steam and combined current-steam and vacuum-formalin disinfectors designed by Dr. M. J. Oliver and Dr. W. Robertson, respectively Medical Officers of Health of Roxburghshire and Selkirkshire and Leith, in conjunction with Mr. John Simpson, an engineer of Stirling. Although those who favour a low-pressure system of steam disinfection can be suited by the adaptability of the "Velox" apparatus, the brochure points out that such a system will not infrequently be found to give an insufficiently high temperature in the chamber to secure disinfection.

### MILK FOR THE "POILU."

In an interesting brochure*, M. Lassablière, chief assistant at the Paris Faculty of Medicine, makes an eloquent plea for the inclusion of condensed milk in the rations issued to troops in the front line of trenches. Compared with that of our own troops, the diet of the French soldier must seem somewhat lacking in sustenance. In practice, as M. Lassablière points out, much of it is wasted, either because of the faulty methods of preparation, or because it does not correspond to the diet to which the peasantry are accustomed. Thus the meat issued totals 800 grammes (approximately 1¾ lb.), an amount altogether disproportionate to the rest of the diet; this results in the men throwing away their rations or distributing them to the villagers. Moreover, the Frenchman is never a big meat-eater; the country folk, like our own, rarely taste it more than once a week, and there have been many cases of enteritis and renal trouble due to this too exclusively carnivorous diet.

The calorie value of the whole ration issued amounts to only 3,189.7; a total which compares poorly with that of our own men, which is well over 5,000.

On the English front six million tins of condensed milk are issued monthly, in addition to a quantity sold in the canteens in the form of cocoa or *café-au-lait*. At present the *poilu* in the front line receives no milk at all; his beverage consists of coffee, twice daily, and half a pint of red wine with the midday meal. In rest billets a certain amount of fresh milk is obtainable, but, owing to the impossibility of any adequate sanitary supervision, much of it is tainted, and a positive danger to those who consume it.

* *Le Lait condensé en campagne.* A. Maloine et Fils. Paris. 1917.

